# Potency Testing of Venoms and Antivenoms in Embryonated Eggs: An Ethical Alternative to Animal Testing

**DOI:** 10.3390/toxins13040233

**Published:** 2021-03-24

**Authors:** Erin E. Verity, Kathy Stewart, Kirsten Vandenberg, Chi Ong, Steven Rockman

**Affiliations:** 1 Technical Development, Seqirus Ltd., Parkville, VIC 3052, Australia; erin.verity@seqirus.com (E.E.V.); kathy.stewart@seqirus.com (K.S.); kirsten.vandenberg@seqirus.com (K.V.); chi.ong@seqirus.com (C.O.); 2 Department of Microbiology and Immunology, University of Melbourne, Parkville, VIC 3052, Australia

**Keywords:** embryonated eggs, venom, antivenom, potency, neutralization

## Abstract

Venoms are complex mixtures of biologically active molecules that impact multiple physiological systems. Manufacture of antivenoms (AVs) therefore requires potency testing using in vivo models to ensure AV efficacy. As part of ongoing research to replace small animals as the standard model for AV potency testing, we developed an alternate in vivo method using the embryonated egg model (EEM). In this model, the survival of chicken embryos envenomated in ovo is determined prior to 50% gestation, when they are recognized as animals by animal welfare legislation. Embryos were found to be susceptible to a range of snake, spider, and marine venoms. This included funnel-web spider venom for which the only other vertebrate, non-primate animal model is newborn mice. Neutralization of venom with standard AV allowed correlation of AV potency results from the EEM to results from animal assays. Our findings indicate that the EEM provides an alternative, insensate in vivo model for the assessment of AV potency. The EEM may enable reduction or replacement of the use of small animals, as longer-term research that enables the elimination of animal use in potency testing continues.

## 1. Introduction

Venoms are complex mixtures of biologically active molecules, that have the potential to impact multiple physiological systems. Envenomation of humans is treated by administration of antivenoms (AVs), typically consisting of purified polyclonal IgG (or fragments) derived from animals immunized with one or more venoms. In Australia, Seqirus Ltd. (CSL) manufactures AVs for humans envenomated with snake, spider, and marine venoms. These venoms include a broad array of toxic components that must be neutralized for the AVs to be effective ([1,2,3,4,5,6,7,8,9,10,11,12], summarized in Table 1).

Due to the complex nature of venoms, the potency of both venoms and AVs is typically tested in small animals, as recommended by the World Health Organization (WHO, [13]). Recommended testing includes the 50% lethal dose (LD_50_) to determine venom potency, and the 50% effective dose (ED_50_) to determine the neutralizing capacity of AVs.

The use of small animals for venom and AV potency testing is of increasing ethical concern. Scientists and AV manufacturers are encouraged to apply the ‘3Rs’—replacement, reduction, and refinement of animal testing. Ethical guidelines may limit the testing that may be performed and the numbers of animals used, which may impact test results. In Victoria, Australia, the use of animals for scientific studies is regulated by the Prevention of Cruelty to Animals (POCTA) Act and other legislation [14,15]; similar legislation is in place in most other jurisdictions. Hence, alternative methods for venom and AV potency testing are required.

The current study investigates the feasibility of testing in an insensate in vivo model, the embryonated egg model (EEM). In the EEM, embryonated chicken eggs are envenomated by injection of venom into the egg albumen, and embryo viability is determined by candling. Embryos are euthanized prior to 50% gestation, at which point the neural system is not considered sufficiently developed for the embryo to experience pain [16,17]. As they are considered insensate, embryos below 50% gestation do not meet the Victorian statutory definition of an animal and are excluded from the Victorian animal welfare legislation [14,15] and the *Australian code for the responsible conduct of research* [14]. Use of such embryos also complies with the 2020 American Veterinary Medical Association (AVMA) guidelines [18], which amended the period avian embryos are considered to be insensate from the first 50% to the first 80% of gestation.

The use of chick embryos for testing of pharmaceuticals is not new [19,20,21,22,23]. In the field of venomics, Sells et al. developed a method for testing the hemorrhagic effect of snake venoms on chick embryos, as an alternative to the WHO-approved rodent intradermal skin test for assessing venom neutralization [24,25,26]. In this assay, paper discs loaded with venom (with or without incubation with AV) were placed onto yolk sac vitelline veins of 6-day old shell-less, ex-ovo chick embryos, which were observed up to 6 h post-envenomation for cessation of heartbeat. This assay was found to correlate well with rodent assays for testing the potency and neutralization of non-neurotoxic snake venoms [24,25]. However, the assay could not be used for neurotoxic venoms due to the incomplete neural arcs in embryos at this early stage of development, or for longer-acting toxins due to the short timeframe of the assay.

Hence, the current study details the development of an alternate in ovo assay for assessing venom and AV potency.

## 2. Results

### 2.1. Feasibility of the Embryonated Egg Model

Initial experiments were performed based on the ex-ovo embryo model of Sells et al. [26], by envenomation of 6-day-old ex-ovo embryos with paper discs loaded with 0.0, 0.2, or 2.0 µg of tiger snake venom. As Australian elapid snake venom may contain high levels of neurotoxins (Table 1), embryos were incubated until they were 10 days old. Of the embryos envenomated with 2 µg/embryo, 25% were non-viable by day 8, and 50% by day 9, suggesting that longer-acting toxins, or toxins acting on later-developing physiological systems, were able to be detected in the chick embryo model.

However, we found that preparation of large numbers of ex-ovo embryos was time consuming, and there was a high background mortality rate.

**Figure 1:**
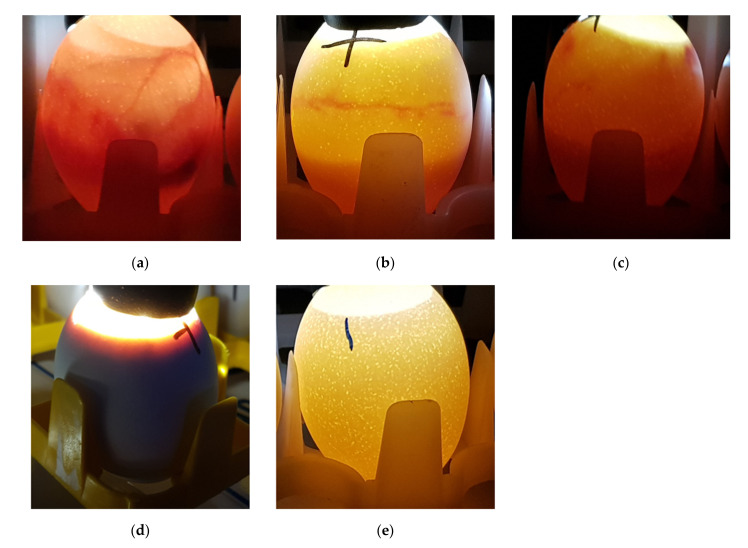
Egg viability on day 10 of gestation. (**a**) Viable egg; (**b**,**c**) Non-viable eggs; (**d**) Opaque egg (non-viable); (**e**) Infertile egg.

Therefore, we tested the susceptibility of embryos in ovo by direct injection of venom into the egg through a small hole placed in the shell in the air sac. As white shelled eggs were used, viability was easily determined by candling early on day 10 of gestation (Figure 1). Eggs were placed at −80 °C by 235 h post-setting, ensuring euthanasia prior to 50% gestation (day 10.5, 242 h post-setting).

Eggs were envenomated in this manner using selected doses of representative venoms used at Seqirus Ltd. (Table 2). All of the venoms tested caused 100% mortality at the highest dose tested except for BJF venom, which caused 75% mortality.

To assess repeatability, the LD_50_ of taipan venom (lot TP355) was measured in six separate assays, and was found to be 0.99 ± 0.41 µg/egg (range 0.61 to 1.50 µg/egg). However, for some assays, the mortality of negative control embryos inoculated using PBS alone was high (up to 33.3%).

Therefore, an alternative method of envenomation of eggs was investigated, by injection of venom using a short (0.5 inch) needle through a small hole placed in the shell below the chorioallantoic membrane (CAM). Injection using this side envenomation method reduced the potential for trauma to the CAM compared to the top envenomation method, reducing the background mortality in control eggs injected with PBS or AV alone in triplicate comparison assays (average 16.1% mortality for top envenomation method compared to 12.5% mortality for side envenomation method).

These results indicate the feasibility of the EEM to assess the activity of a range of snake, spider, and marine venoms.

### 2.2. Measurement of AV Potency

#### 2.2.1. Optimization of Venom Test Dose

Antivenom potency was measured in the EEM by incubation of a constant dose of venom (the venom test dose, VTD; µg/egg) with graded dilutions of AV, prior to injection into eggs using the side inoculation method. The VTD must be sufficient to consistently render 100% of test embryos non-viable in the absence of AV, however a very high VTD requires large volumes of venom and AV reagents. In small animal assays at Seqirus Ltd. a venom dose ≥3 LD_50_/animal is used, and the WHO recommend a venom dose of 3–6 LD_50_/animal [13]. In the EEM assay developed here, this number is expressed as the venom lethal dose (VLD, LD_50_/egg), and is calculated from the result of LD_50_ assays performed at the same time as the ED_50_.

**Figure 2:**
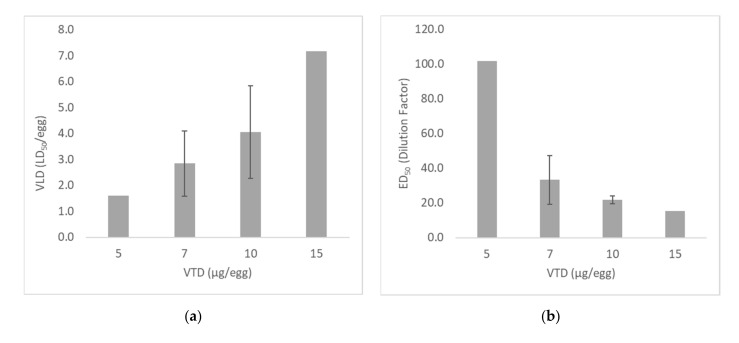
(**a**) Correlation of VTD (µg/egg) with VLD (LD_50_/egg) for measurement of taipan venom; (**b**) correlation of VTD with ED_50_ (AV dilution factor) for measurement of neutralization of taipan venom with four Taipan or Polyvalent AVs. Results were compiled from four separate comparative assays in the embryonated egg model (VTD of 5 µg/egg, *n* = 1 result; 7 µg/egg, *n* = 4; 10 µg/egg, *n* = 4; 15 µg/egg, *n* = 2). Error bars represent one standard deviation.

The ED_50_ of four AV lots was measured in separate assays, demonstrating a linear relationship between the VTD and VLD (Figure 2). The LD_50_ of the taipan venom was measured as 3.0–3.5 µg/egg for all assays except assay C (1.5 µg/egg), resulting in higher VLDs for this experiment. A VTD of 5 µg/egg was only tested in a single assay as the resulting VLD was very low (1.6 LD_50_/egg), but has been included to demonstrate the trend that was observed. For a VTD of 10 µg/egg, the VLD ranged from 2.9 to 6.7 LD_50_/egg.

Increasing the VTD resulted in lower AV ED_50_ dilution factors, as more AV was required to neutralize higher venom doses (Figure 2). When tested in guinea pigs (GPs), the potency of the four AV lots tested was relatively similar (range 261–345 U/mL), which was reflected using a VTD of 10 µg/egg.

Together, these results indicate the importance of adjusting the VTD used so that the VLD is ≥3 LD_50_/egg. For taipan venom under the conditions used for the assays shown in Figure 2, the optimum VTD was ≥10 µg/egg.

#### 2.2.2. Optimization of Egg Age and Venom/AV Incubation Conditions

Typical venom neutralization assays include a step for incubation of venom with AV, to allow antibody binding prior to injection of a test animal. The WHO guidelines recommend incubation for 30 min at room temperature (RT) [13].

The effect of incubation temperature was investigated by testing AV potency against taipan venom, and calculating the AV potency (U/mL, where one unit is the AV volume that 50% neutralized 10 µg of venom). As the potency of each AV lot in the GP model was known, the AV potency recovery for each assay was calculated as the % potency in the EEM compared to the GP model. The AV potency recovery was used to directly compare results for different assays and different AV lots.

**Figure 3:**
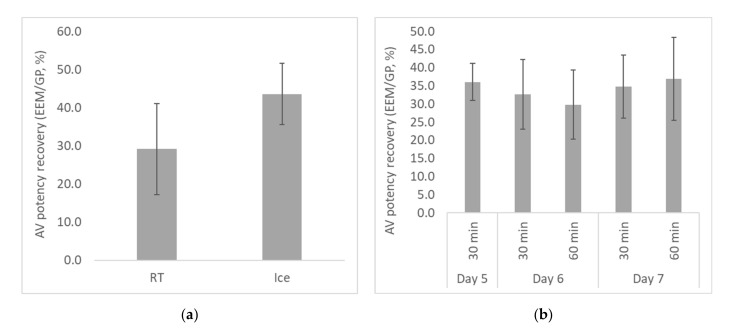
Effect of incubation conditions on AV potency recovery, measured using taipan venom. (**a**) Venom and AV were incubated for 30 min at RT or on ice prior to injection of eggs (*n* ≥ 5); (**b**) Venom and AV were incubated on ice for 30 or 60 min, prior to injection of eggs on day 5, 6, or 7 of gestation (*n* ≥ 4). The AV potency recovery was calculated as the % potency in the embryonated egg model (EEM) compared to guinea pigs (GP).

First, the effect of incubation temperature was investigated by incubating the venom/AV mixture for ≥30 min, either at RT (*n* = 5 separate experiments) or on ice (*n* = 6). The AV potency recovery was higher when the incubation step was performed on ice (Figure 3a), although the difference was not quite significant (*p* = 0.056). The mechanism for this is unclear, although it is possible that some degradation of the venom occurred during the room temperature incubation. Should this be the case, modification of the assay to perform the venom/AV incubation on ice may increase the accuracy of AV potency measured in the EEM. However, this should be confirmed in specific optimization studies for each venom to be tested.

The effect of incubation time and egg age was then investigated by incubating the venom/AV mixtures on ice for either 30 or 60 min, prior to incubation of eggs on day 5, 6, or 7 of gestation. The conditions tested were: 30 min incubation, day 5 eggs (*n* = 4); 30 min incubation, day 6 eggs (*n* = 29); 60 min incubation, day 6 eggs (*n* = 5); 30 min incubation, day 7 eggs (*n* = 10); and 60 min incubation, day 7 eggs (*n* = 8).

The results demonstrated that the AV potency recovery was not significantly affected by either the incubation time or the egg age between days 5 to 7 of gestation (Figure 3b). However, for some eggs it was difficult to determine egg viability on day 5 of gestation. For 7-day-old eggs, the larger size of the CAM may increase the risk of trauma to the CAM during the injection process, although there was no significant difference in the mortality rate of control eggs injected with PBS or AV alone (9.3 ± 8.1% for day 6 eggs; 5.9 ± 10.3% for day 7 eggs). For 28 separate assays, the mortality rate for control groups ranged from 0.0 to 29.4% (mean 7.9%, median 6.7%), with no unexpected mortality of embryos in LD_50_ or ED_50_ groups. Assays with high negative control mortality rates typically had low numbers of eggs in negative control groups. These data suggest that mortality of >30% in small PBS or AV control groups is required to indicate excess mortality due to external factors.

From these results, incubation conditions of 30 min on ice, and injection of eggs on day 6 of gestation, were selected for further development of the EEM.

#### 2.2.3. Optimization of Venom Potency by Addition of BSA

Venoms are complex mixtures containing various proteins and peptides, which in some instances have a tendency to adhere to glassware [27]. The potency of some venoms may increase when 0.1% bovine serum albumin (BSA) is used for venom dilution, to block adherence of venom peptides to glass [28]. The use of 1% BSA for this purpose is suggested by the WHO for analysis of manufactured AVs [13]. To assess the potential impact of venom protein loss on venom and AV potency in the EEM, dilution of taipan venom and AV was performed using 0.1% BSA diluent, in pre-rinsed glass bottles.

**Figure 4:**
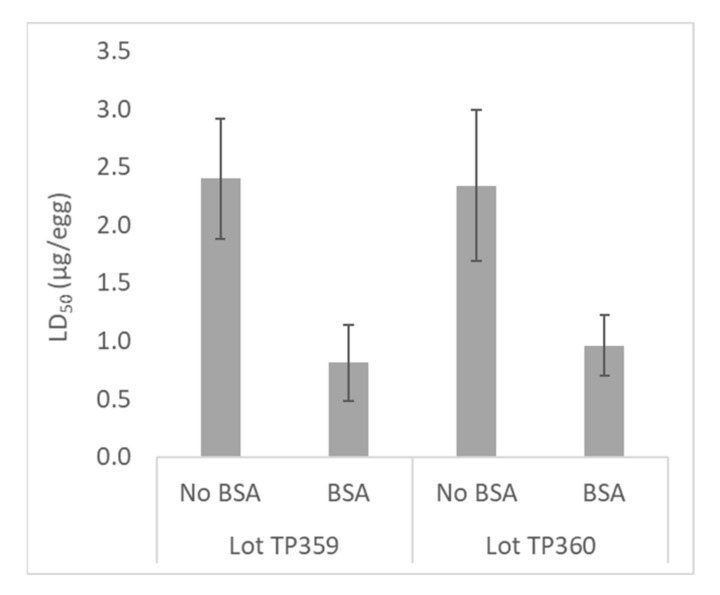
Effect of bovine serum albumin (BSA) on potency of taipan venom and AV. Two lots of taipan venom were diluted using 0.1% BSA prior to measurement of LD_50_ in the embryonated egg model (EEM) (*n* = 3–5; left).

The use of 0.1% BSA for venom dilution consistently increased venom potency, reflected in the decreased LD_50_ result for two lots of taipan venom in the presence of BSA (Figure 4). The difference in LD_50_ in the presence of BSA was significant (*p* = 0.018 for lot TP359; *p* = 0.006 for lot TP360).

### 2.3. Performance of the EEM Assay

#### 2.3.1. Measurement of AV Potency

**Figure 5:**
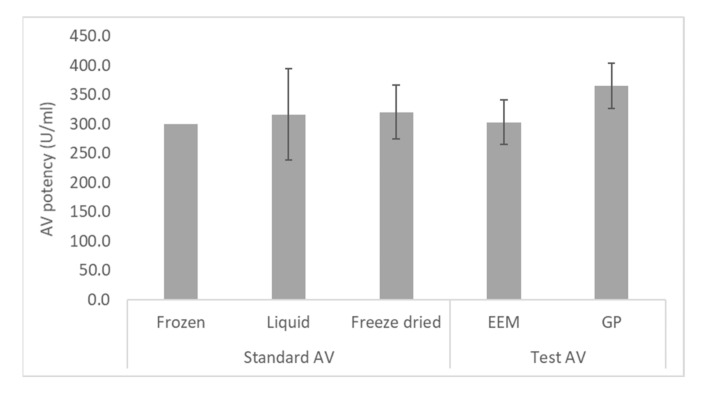
Measurement of the potency of three standard AVs stored in different formats and a test AV (lot 0555 20001), of known potency in the guinea pig (GP) model. The embryonated egg model (EEM) was used to measure the ED_50_ of the standard AVs in quadruplicate, and the test AV in triplicate. For each assay, the potency of each AV was calculated relative to the frozen standard AV (prepared at 300 U/mL using potency of 333 ± 36.2 U/mL in the GP model).

The EEM was used to measure the potency of a standard AV stored in three formats (liquid at 2–8 °C, frozen at −80 °C, and freeze dried, stored at −80 °C), as well as a test AV (lot 0555 20001), each of known potency in the GP model. When the potency of the liquid and freeze-dried standard AVs were calculated relative to the frozen standard AV, the average potency measured in the EEM was within 7% of potency of the frozen standard AV (Figure 5), and the potency differences were not significant (*p* = 0.705 for the liquid standard AV; *p* = 0.435 for the freeze dried standard AV). This result indicates that the storage conditions of the standard AV did not significantly impact the measured potency. When the potency of the test AV was measured in the EEM relative to the frozen standard AV, it was within 17% of the potency measured in the GP model (Figure 5). The difference in potency was not significant (*p* = 0.089), demonstrating the potential of the EEM for measurement of AV potency.

To assess assay specificity, taipan venom was incubated with graded dilutions of polyvalent Taipan AV or heterologous Stonefish AV prior to injection of eggs. While the taipan venom was neutralized by the Taipan AV (ED_50_ dilution factor, 24), 100% mortality of envenomated embryos was observed for all Stonefish AV dilutions (dilution factors tested ≥ 4), demonstrating that the neutralization of venom observed using the EEM was specific.

#### 2.3.2. Breadth of Venom Activity in the EEM

To assess the breadth of venom activity in the EEM, the LD_50_ of elapid snake, spider, and marine venoms used at Seqirus Ltd. was measured in the EEM, except for redback spider for which insufficient venom was available for testing. All of the venoms tested caused mortality of the chick embryos in a dose-dependent manner (Table 3). There was not a clear relationship for venom potency in the EEM compared to small animal models, as the LD_50_ (mg/kg) for some venoms in the EEM was similar (taipan, tiger snake, sea snake), higher (brown snake, box jellyfish), or lower (black snake, death adder, funnel-web spider, stonefish) than the LD_50_ in small animal models.

All of the venoms tested were active in the EEM, including those with significant neurotoxic, pore-forming toxin, cardiotoxic, and myotoxic components. Importantly, this included FWS venom, which is poorly toxic for vertebrates other than primates and newborn mice [29,30]. Eggs envenomated with FWS venom were often opaque in appearance, as shown in Figure 1.

Hence, these results demonstrate the potential of the EEM for measuring the potency of snake, spider, and marine venoms and AVs. A broader range of venoms and purified toxins are to be analysed in further studies, to better understand the mechanisms active in the EEM and the universality of the model.

## 3. Discussion

In this study, an alternative, insensate in vivo model was developed for the measurement of venom and AV potency. Using a standard AV of known potency, the potency of a test AV measured against taipan venom using the EEM correlated with that measured using a small animal assay.

For AV manufacture, potency testing of venoms and AVs is typically performed in small animals [13] under the guidance of institutional animal ethics committees (AECs) and according to local animal welfare legislation. Often, the ability to perform any non-critical testing is limited; however, the EEM assay developed here may have broader application including developmental and investigative studies, since the embryos used are euthanized prior to 50% gestation. The embryos do not meet the Australian statuary definition of an animal and hence, are not subject to animal welfare legislation.

The use of chicken embryos for assessment of venom and AV potency has been studied previously. Sells et al. [24,25,26] developed a shell-less, ex-ovo chicken embryo model for measurement of LD_50_ and ED_50_ for hemorrhagic snake venoms, where the mortality of 6-day-old embryos was assessed 6 h post-envenomation. The ex-ovo assay was reported to have low efficacy for venoms that were primarily neurotoxic, such as venom from elapid snakes which are of primary clinical significance in Australia.

Unlike the ex-ovo assay, all of the venoms used at Seqirus Ltd. were active in eggs, regardless of primary mode(s) of action. The ability to test FWS venom in the EEM is particularly important, since the only vertebrate species known to be highly susceptible to FWS venom are primates (including humans) and newborn mice [29,30]. It is possible that the activity of some venom components differs between the EEM and mammalian animal models, although no other evidence for this was observed in this study. Future studies analyzing venoms from additional species, as well as purified individual toxins, will help elucidate the activity of different venom components in the EEM.

For some venoms, potency measured in the EEM differed slightly from that measured in animal models. This was not surprising, as the variability of venom LD_50_ measurement is well documented, and depends on a number of factors including the test animal species and route of delivery [28,31,32,33,34,35]. In addition, the longer incubation time for the EEM assay may allow venom components with longer acting times to have greater impact. The addition of 0.1% BSA to the venom/AV diluent resulted in increased taipan venom potency, as has been observed previously [28], and likely resulted from blocking the adherence of venom proteins to the glass vessels used to prepare venom doses [27,36].

Except for BJF, envenomation of humans typically occurs via puncture wounds in the skin, most often on limbs [37]—similar to subcutaneous (SC) injection. The side inoculation method used in the EEM is similar to SC inoculation, as the venom is injected below the CAM into the egg albumin, from where it can disseminate throughout the egg. Locating the CAM by candling six-day-old eggs was found to be simple when white-shelled eggs were used.

The use of large numbers of embryos inevitably resulted in the loss of some embryos in negative control groups during incubation, which likely resulted from natural attrition, trauma, or contamination caused by the injection process. It was noted that mortality rates for embryos inoculated with sub-lethal doses of venom were often lower than for negative control groups, where mortality rates could be up to 30%. Mortality rates >30% in negative control groups generally indicated unfavorable incubation conditions. Therefore, we propose that a mortality rate of ≤ 30% in small negative control groups is tolerable, and did not appear to affect assay results.

Prior to use of the EEM for AV manufacture, further studies are required to optimize and validate the assay for each venom. Optimization could include venom and AV dilution, the use of BSA, the use of a standard AV of known potency, and confirmation of the limit for mortality rate for eggs in negative control groups injected with PBS or AV alone. Further studies investigating the activity of additional venoms and purified toxins will aid elucidation of the mechanisms active in the EEM and the universality of the model.

Together, these results demonstrate the feasibility of testing venom and AV potency in the EEM developed in this study. This would result in minimization, and potentially replacement, of the small animal assays currently in use for measurement of venom and AV potency.

## 4. Materials and Methods

### 4.1. Venoms, AVs, and Eggs

Venoms and AVs (bulk concentrate and finished product) were obtained from the Antivenoms/Antitoxins (AVAT) Manufacturing department at Seqirus Ltd. (Parkville, Victoria, Australia), and are summarized in Table 4. Lot numbers were included in the results sections where relevant.

Bulk venoms were obtained as described in Table 4, and were stored lyophilized. After reconstitution in 0.9% saline, venoms were stored at −80 °C in single-use aliquots. Venoms were inoculated into Luria broth to check sterility.

Antivenoms were manufactured by Seqirus Ltd. in horses, rabbits, or sheep (Table 4), and were stored at 2–8 °C. Antivenoms raised against the land snakes (black, taipan, death adder, tiger, and brown snakes) were polyvalent for all of the land snakes.

As part of the AV manufacturing process, the potency of all venoms and AVs was tested in small animals (Table 4) by the Seqirus Ltd. AVAT Manufacturing and QC departments.

The standard AV of known potency was prepared from polyvalent AV lot 0555 20301 (GP potency 333 ± 36.2 U/mL), diluted to 300 U/mL using PBS. The standard AV was stored in single-use aliquots at 2–8 °C, −80 °C, or freeze-dried at 150 U/mL in the presence of 3% dextran, and stored at −80 °C. The freeze-dried standard AV was reconstituted at 300 U/mL using water for injection (WFI).

Specific-pathogen free (SPF) eggs were obtained from Australian SPF Services Pty Ltd. (Woodend, Victoria, Australia). Fertilized eggs were stored for one day at 18 °C prior to setting at 37 °C.

### 4.2. Assessment of Venom Potency (LD_50_)

Assessment of venom LD_50_ in the EEM was performed by serial dilution of venoms using PBS (with or without 0.1% BSA, as specified), using dilution factors of 2 (taipan) or 5 (sighter assays for remaining venoms to qualitatively determine efficacy in the EEM). For each assay, 4–7 venom dilutions were tested. Venoms and dilutions were stored on ice until envenomation of 5–7-day-old eggs (0.2 mL/egg).

**Figure 6:**
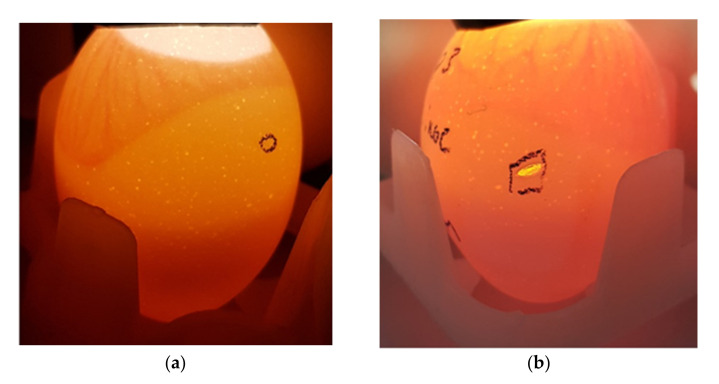
(**a**) Location of injection of embryonated eggs below the CAM (5-day-old egg shown); (**b**) Hole placed into the shell for injection, leaving the shell membrane intact.

For each venom dilution, 4 viable eggs were envenomated using the top injection method (using a 1-inch needle through a small hole punched in the air sac at the top of the egg) or using the side injection method. For the side method, eggs were candled to locate the CAM (Figure 6a), and a Dremel Stylo+ tool with grinding wheel attachment was used to make a hole in the shell below the CAM, leaving the shell membrane intact (Figure 6b). A hole was also made in the shell in the air sac to allow for membrane expansion.

For envenomation, eggs were placed on an approximate 45° angle, and a syringe fitted with a 0.5-inch needle was used to deliver a 0.2 mL dose through the exposed shell membrane. The hole was sealed using electrical tape, and the eggs were incubated at 37 °C. In each assay, a control group of ≥4 eggs were injected with PBS.

Early on day 10 of gestation (prior to 235 h post-setting), eggs were candled to determine viability (Figure 1). Viable eggs were defined as those having both blood vessels and an embryo clearly visible. Viable eggs were euthanized by placing them at −80 °C prior to 235 h post-setting, to ensure that euthanasia occurred prior to 50% gestation (252 h post-setting). Eggs were stored at −80 °C for ≥24 h.

Venom potency (LD_50_) was calculated using Combistats software (Version 6; European Directorate for the Quality of Medicines and Healthcare (EDQM), Strasbourg, France) using Probit or Spearman-Karber methods.

Statistical significance was determined using a Student’s two-tailed *t*-test.

### 4.3. Assessment of AV Potency (ED_50_)

Assessment of AV ED_50_ in the EEM was performed by serial twofold dilution of AV, and incubation with a constant venom dose (the venom test dose, VTD). Unless otherwise stated, the incubation was performed for 30 min on ice, prior to envenomation of eggs using the top or side envenomation methods as described for assessment of venom LD_50_. Venom and AV were diluted using PBS (with or without 0.1% BSA, as specified). Venoms were stored on ice for the duration of the assay prior envenomation of 5–7-day-old eggs (0.2 mL/egg).

For each assay, 4–6 AV dilutions were tested, using 4–6 eggs per dilution. A group of control eggs (≥4) was injected with the lowest AV dilution tested, in the absence of venom.

For each assay, the number of lethal venom doses received by each egg (the venom lethal dose, VLD), was calculated as the VTD (µg/egg)/LD_50_ (µg/egg), using the results of concurrent LD_50_ assays. Dose-finding assays were used to calibrate the VTD so that the VLD was ≥3 LD_50_/egg, where possible. The AV dilution range was then calibrated to the VTD.

Early on day 10 of gestation, eggs were candled to determine viability as described for the LD_50_ assay, and viable eggs euthanized by freezing prior to 50% gestation.

For each assay, the ED_50_ (measured as the AV dilution factor to cause 50% neutralization of venom) was calculated using Combistats software using the Probit or Spearman-Karber methods.

Antivenom potency was dirently calculated in units/mL (where a unit is the AV volume calculated to neutralize 10 µg of venom) using Equation (1) (using VTD and LD_50_ in mg/egg, and 0.2 mL/egg injection volume).

(1) $$\mathrm{AV}\;Potency\;\left(U/mL\right)\;=100\times \frac{\mathrm{VTD}-{\mathrm{LD}}_{50}}{\left(0.2/{\mathrm{ED}}_{50}\right)}$$

Potency of a test AV, relative to a standard AV of known potency tested at the same time, was calculated using Equation (2).

(2) $$Test\;\mathrm{AV}\;potency\;\left(U/mL\right)\;=Standard\;\mathrm{AV}\;potency\;\left(U/mL\right)\times \frac{Test\;{\mathrm{ED}}_{50}}{Std\;{\mathrm{ED}}_{50}}$$

Statistical significance was determined using a Student’s two-tailed *t*-test.

## Figures and Tables

**Table 1:** Venoms used at Seqirus Ltd. and their primary toxic components.

		Neurotoxins						Coagulants	
Common Name	Species	α- ^1^	β- ^2^	Other	PFT ^3^	CT ^4^	MT ^5^	Pro-	Anti-
Black snake (King Brown/Mulga)	*Pseudechis australis*	+	+				+		+
Coastal taipan	*Oxyuranus scutellatus*	+	+				+	+	
Death adder	*Acanthophis antarcticus*	+	+						+
Tiger snake	*Notechis scutatus*	+	+				+	+	
Eastern brown snake	*Pseudonaja textilis*	+	+					+	
Sea snake	*Enhydrina schistosa*	+					+		
Funnel-web spider (FWS)	*Atrax robustus*			+					
Redback spider (RBS)	*Latrodectus hasselti*		+		+				
Box jellyfish (BJF)	*Chironex fleckeri*			+	+	+	+		
Stonefish (SF)	*Synanceia sp.*		+	+	+	+	+		

^1^ Post-synaptic; ^2^ pre-synaptic; ^3^ pore-forming toxins; ^4^ cardiotoxins; ^5^ myotoxins.

**Table 2:** Envenomation of chick embryos in ovo with selected doses of representative venoms, using the top envenomation method.

Venom	Susceptibility of Chick Embryos in ovo	Venom Dose Causing 100% Mortality (µg/egg)
Black snake	+	46
Taipan	+	4
Death adder	+	26
Tiger snake	+	4
Sea snake	+	20
Funnel-web spider	+	2
Box jellyfish	+	37.5 U/egg ^1^

^1^ 75% Mortality at a venom dose of 37.5 U/egg.

**Table 3:** Activity of venoms used at Seqirus Ltd. in the embryonated egg model (EEM), by titration using 0.1% BSA. Calculation of LD_50_ (mg/kg) was performed using average egg weight (measured in each assay) for the EEM, and representative weights (GP: 400 g; mouse: 20 g; suckling mouse: 2 g) for small animals. For each venom, the result of a single assay is shown.

		EEM		Small Animal Assay	
Venom	Venom lot	LD_50_ (µg/egg)	LD_50_ (mg/kg)	LD_50_ (µg/animal)	LD_50_ (mg/kg)
Black snake	PA284	0.24	0.0043	295.4	0.739
Brown snake	BR262	6.00	0.1105	6.9	0.017
Death Adder	DA416	0.89	0.0161	60.7	0.152
Taipan	TP359	0.74	0.0134	5.4	0.014
Tiger snake	286048	0.35	0.0063	6.3	0.016
Sea snake	VS0004	5.00	0.0943	23.3	0.058
Funnel-web spider	FW123	4.47	0.0808	3.8	1.900
Redback spider	BO057/ BO058	NR ^1^	NR ^1^	1.0 sac/mouse	50.0 sacs/mouse
Box jellyfish	BJF-020206	37.5 U/egg	678.1 U/kg	1.7 U/mouse	85.0 U/kg
Stonefish	266344	5.2	0.0956	16.7	0.835

^1^ No result (NR) as insufficient venom was available for testing in the EEM.

**Table 4:** Venom, AV, and potency testing at Seqirus Ltd.

Antivenom Type	Venom Species	Venom Supplier	Species AV Raised in	Species AV Tested in
Black snake	*Pseudechis australis*	ARP ^1^	Horses	Guinea pigs
Taipan	*Oxyuranus scutellatus*	ARP ^1^	Horses	Guinea pigs
Death Adder	*Acanthophis antarcticus*	ARP ^1^	Horses	Guinea pigs
Tiger Snake	*Notechis scutatus*	ARP ^1^	Horses	Guinea pigs
Brown Snake	*Pseudonaja textilis*	ARP ^1^	Horses	Guinea pigs
Polyvalent Snake	All above	As Above	Horses	Guinea pigs
Sea Snake	*Enhydrina schistosa*	VS ^2^	Horses	Guinea pigs
Funnel-Web Spider	*Atrax robustus*	ARP ^1^	Rabbits	Suckling mice
Redback Spider	*Latrodectus hasselti*	VS ^2^	Horses	Mice
Box Jellyfish	*Chironex fleckeri*	Vision ^3^	Sheep	Mice
Stonefish	*Synanceia horrida, S. verrucosa*	JCU ^4^	Horses	Mice

^1^ Australian Reptile Park, Somersby, NSW, Australia; ^2^ Venom Supplies Pty Ltd., Tanunda, SA, Australia; ^3^ Vision Pty; ^4^ James Cook University, QLD, Australia.

## Data Availability

The data presented in this study are available on request from the corresponding author.
